# Correcting time‐intensity curves in dynamic contrast‐enhanced breast MRI for inhomogeneous excitation fields at 7T

**DOI:** 10.1002/mrm.28147

**Published:** 2019-12-27

**Authors:** Michael J. van Rijssel, Josien P. W. Pluim, Hui‐Shan M. Chan, Lieke van den Wildenberg, Alexander M. Th. Schmitz, Peter R. Luijten, Kenneth G. A. Gilhuijs, Dennis W. J. Klomp

**Affiliations:** ^1^ Center for Image Sciences UMC Utrecht Utrecht The Netherlands; ^2^ Department of Biomedical Engineering Technische Universiteit Eindhoven Eindhoven The Netherlands; ^3^ Department of Radiology Onze Lieve Vrouwe Gasthuis Amsterdam The Netherlands

**Keywords:** 7T, $B_{1}^{+}$ mapping, breast, DCE‐MRI, flip‐angle correction, RF field inhomogeneity

## Abstract

**Purpose:**

Inhomogeneous excitation at ultrahigh field strengths (7T and above) compromises the reliability of quantified dynamic contrast‐enhanced breast MRI. This can hamper the introduction of ultrahigh field MRI into the clinic. Compensation for this non‐uniformity effect can consist of both hardware improvements and post‐acquisition corrections. This paper investigated the correctable radiofrequency transmit ($B_{1}^{+}$) range post‐acquisition in both simulations and patient data for 7T MRI.

**Methods:**

Simulations were conducted to determine the minimum $B_{1}^{+}$ level at which corrections were still beneficial because of noise amplification. Two correction strategies leading to differences in noise amplification were tested. The effect of the corrections on a 7T patient data set (*N* = 38) with a wide range of $B_{1}^{+}$ levels was investigated in terms of time‐intensity curve types as well as washin, washout and peak enhancement values.

**Results:**

In simulations assuming a common amount of T_1_ saturation, the lowest $B_{1}^{+}$ level at which the SNR of the corrected images was at least that of the original precontrast image was 43% of the nominal angle. After correction, time‐intensity curve types changed in 24% of included patients, and the distribution of curve types corresponded better to the distribution found in literature. Additionally, the overlap between the distributions of washin, washout, and peak enhancement values for grade 1 and grade 2 tumors was slightly reduced.

**Conclusion:**

Although the correctable range varies with the amount of T_1_ saturation, post‐acquisition correction for inhomogeneous excitation was feasible down to $B_{1}^{+}$ levels of 43% of the nominal angle in vivo.

## 1 INTRODUCTION

The most used sequence in breast MRI is DCE MRI. It has a high diagnostic power because of its ability to detect abnormalities and to differentiate malignant from benign lesions.^1^, ^2^ To a great extent, its diagnostic power is based on differences in dynamics of contrast agent uptake. These differences in contrast agent uptake have led to the characterization of time‐intensity curves (TICs) into 3 categories: type I curves, which show steady enhancement; type II curves, which show a plateau; or type III curves, which show a washout.^3^

Currently, breast MR examinations are routinely performed at field strengths up to 3T. Efforts are underway to enable breast MRI at ultrahigh field strengths, most notably 7T. Advantages of performing MR at ultrahigh field strengths include a higher SNR and a higher chemical shift.^4^ The first advantage can be used to increase spatial resolution, which has been shown to be feasible in a clinical setting and might have potential for earlier and better diagnosis.^5^, ^6^ Additionally, a better spatial resolution enables the assessment of tumor heterogeneity.^7^ The second advantage can be used to measure tumor metabolism using spectroscopy techniques. Measurements of this kind may be able to predict the response to neoadjuvant therapy in an early stage of treatment.^8^, ^9^ A multiparametric analysis combining phosphorous spectroscopy with DCE‐MRI achieves a better agreement with postoperative findings than the conventional preoperative workup.^10^

**Figure 1:**
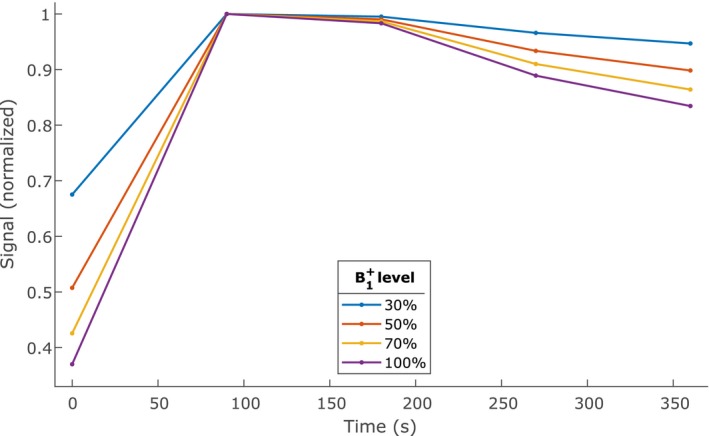
Influence of $B_{1}^{+}$ on time‐intensity curves in DCE‐MRI. Low $B_{1}^{+}$ induces a type II plateau curve, even in tumors that should show a type III washout curve. Each curve has been normalized to its own maximum for clarity

A major factor hampering the clinical acceptance of ultrahigh field breast MRI in the clinic is the fact that time‐intensity curves acquired during DCE MRI acquisitions may be unreliable because of RF transmit ($B_{1}^{+}$) inhomogeneities.^11^, ^12^ These $B_{1}^{+}$ field inhomogeneities increase with increasing field strengths.^4^ A lower $B_{1}^{+}$ level means that spins experience a lower RF excitation angle and consequently a lower amount of T_1_ saturation is applied. For DCE‐MRI, this means that a decrease in $B_{1}^{+}$ level often causes a decrease in sensitivity to changes in T_1_ and ultimately a flattening of TICs, as illustrated in Figure 1.

The most obvious solution may be to tackle the problem at the source: improve the homogeneity of the $B_{1}^{+}$ field. In recent years, progress has been made in the field of RF coil design toward that end.^13^, ^14^ However, a completely homogeneous $B_{1}^{+}$ field is nontrivial to achieve even with state‐of‐the‐art hardware, so some amount of field inhomogeneity is always expected. This is illustrated by recent works showing that corrections for $B_{1}^{+}$ can be beneficial even at 1.5T and 3T.^15^, ^16^

DCE scans can be corrected for inhomogeneous $B_{1}^{+}$ effects post‐acquisition, if the $B_{1}^{+}$ field distribution is known. Haacke et al^17^ described an approach to quantify T_1_ at every time point in a DCE time series that allows straightforward incorporation of $B_{1}^{+}$ field maps to calculate unbiased estimates of T_1_. From these estimates, synthetic MR images corrected for $B_{1}^{+}$ can be generated. However, because this method is based on the ratio of each postcontrast image with the precontrast image, the resulting SNR is limited by the low SNR of the precontrast image. We investigate a simplification of this 2‐step approach to limit the amount of noise amplification in the corrected images.

**Figure 2:**
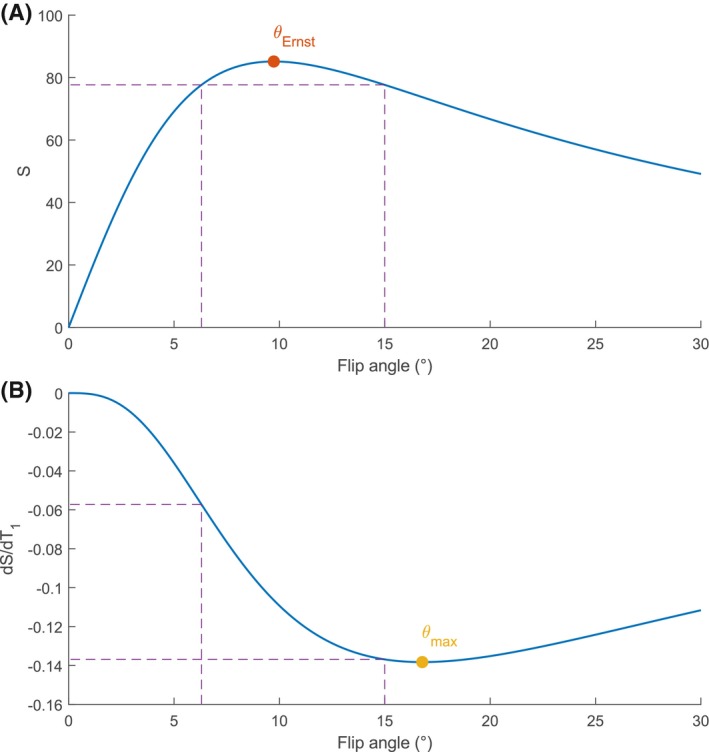
(A) Signal intensity (S) versus flip angle. In case of a nominal imaging angle above the Ernst angle (θ_Ernst_), a lower flip angle can be found that produces the same signal intensity, the equivalent angle. The range between the nominal angle and the equivalent angle determines the $B_{1}^{+}$ buffer in terms of signal loss. (B) Derivative of the signal intensity with respect to T_1_ versus flip angle. The sensitivity to T_1_ changes decreases rapidly for low flip angles. This limits the correctable $B_{1}^{+}$ range further

Even though $B_{1}^{+}$ corrections post‐acquisition are possible, the correctable range will be limited by the absolute amount of signal that is generated at the actual flip angle. Figure 2A shows the amount of signal generated for a range of flip angles by an enhancing tumor, assuming a spoiled gradient echo imaging sequence with a TR of 5.8 ms (used in this study) and enhancing signal from tumor tissue with a T_1_ of 400 ms (roughly based on the average T_1_ of enhancing tissue that was found in this study). If an imaging angle above the Ernst angle is used (e.g., 15° as in this study), there will be a lower angle that generates the same amount of signal; in this example, that is 6.3°. Therefore, one might think that the range of $B_{1}^{+}$ at which correction is still possible extends at least as far as 6.3/15 = 42% of the nominal angle. However, a decrease in flip angle not only induces a change in signal intensity, it also means a change in T_1_ sensitivity. Figure 2B shows the change in signal induced by a change in T_1_. Even though at an imaging angle of 6.3° the amount of generated signal is equivalent, the sensitivity of the signal to changes in T_1_ is only 41.8% of the sensitivity at the nominal angle. This decrease in sensitivity to changes in T_1_, and ultimately to changes in contrast uptake, will further limit the correctable $B_{1}^{+}$ range.

This paper investigates the $B_{1}^{+}$ range in which DCE‐MRI is still correctable post‐acquisition. This will ultimately dictate the degree of homogeneity of the $B_{1}^{+}$ field that coil designers need to achieve. This study will include both simulations and patient data obtained at 7T, which were acquired with a large variation in $B_{1}^{+}$ levels. Furthermore, we will look at the effect of correction on the patient data with respect to individual time‐intensity curves and curve types.

## 2 METHODS

### 2.1 Correction strategies

The measured signal in DCE MRI can be described using the signal equation for spoiled gradient echo acquisitions

(1) $$S\left(\theta \right)=\rho \frac{\sin\left(\theta \right)\left(1-e^{-TR/T_{1}}\right)}{1-\cos\left(\theta \right)\;e^{-TR/T_{1}}},$$

where *S* is the MR signal, θ is the flip angle, ρ is the proton density weighted with the sensitivity of the MR system’s receive chain, TR is the repetition time, and T_1_ is the longitudinal relaxation time.

Two correction strategies were implemented in MATLAB (R2017b, The MathWorks, Natick, MA). The first strategy was based on the T_1_ quantification method of Haacke et al.^17^ Using this method, a T_1_ value at every time point was calculated using $B_{1}^{+}$ information and assuming a fixed T_1_ of glandular tissue. For all patients in the data set, a fixed precontrast T_1_ value was assumed as proposed by Haacke et al.^17^ The value that was used was 2100 ms, which corresponds to the average of a previously measured group of healthy volunteers.^18^ Subsequently, synthetic MR images were calculated using the calculated T_1_ maps, the protocol TR, nominal flip angle, and an estimated ρ‐map. The ρ‐map was estimated using the precontrast image, by inverting the signal equation (Equation 1).

The method of Haacke et al^17^ was devised to achieve quantification of contrast enhancement in DCE MRI. This quantification is achieved by measuring the ratio of the signal intensity at every time point with respect to the precontrast signal intensity, at which the T_1_ is known or assumed. This ratio image can only be reliably calculated if all postcontrast images align well with the precontrast image. Moreover, the SNR of the resulting images is limited by the SNR of the noisiest image in the DCE series, usually the precontrast image. Because the goal of the current study is not to quantify the contrast enhancement, but to correct for $B_{1}^{+}$ inhomogeneity, a more direct approach was devised. This approach circumvents the calculation of the ratio with respect to the precontrast image and skips the T_1_ quantification step.

The proposed method aims to achieve a direct mapping from the measured signal intensities to the true, or corrected, signal intensities using $B_{1}^{+}$ information obtained using the actual flip‐angle imaging method (AFI) (see Section 2.3). Such a mapping can be achieved analytically, starting with the signal equation for spoiled gradient echo acquisitions as described by Equation 1. The measured signal at any $B_{1}^{+}$ level is then given by *S*(*f* θ_nom_), with *f* the $B_{1}^{+}$ fraction and θ_nom_ the nominal flip angle. Therefore, the relationship between measured and true signal intensities can be described using the following system of equations

(2) $$\left\{\begin{array}{c} M=S(f\theta_{\text{nom}})\\ T=S(\theta_{\text{nom}}) \end{array}\right.,$$

where *M* is the measured signal at $B_{1}^{+}$ level *f* and *T* is the true signal at a $B_{1}^{+}$ level of 100%. The system in Equation 2 can be solved for the true signal *T*, which gives a direct mapping from measured signal intensities to true signal intensities

(3) $$T\left(M,\;f\right)=\frac{M\rho \;\sin\left(\theta_{\text{nom}}\right)\left(\cos(f\theta_{\text{nom}})-1\right)}{M\left(\cos\left(f\theta_{\text{nom}}\right)-\cos\left(\theta_{\text{nom}}\right)\right)+\rho \sin\left(f\theta_{\text{nom}}\right)\left(\cos(\theta_{\text{nom}})-1\right)}.$$

**Figure 3:**
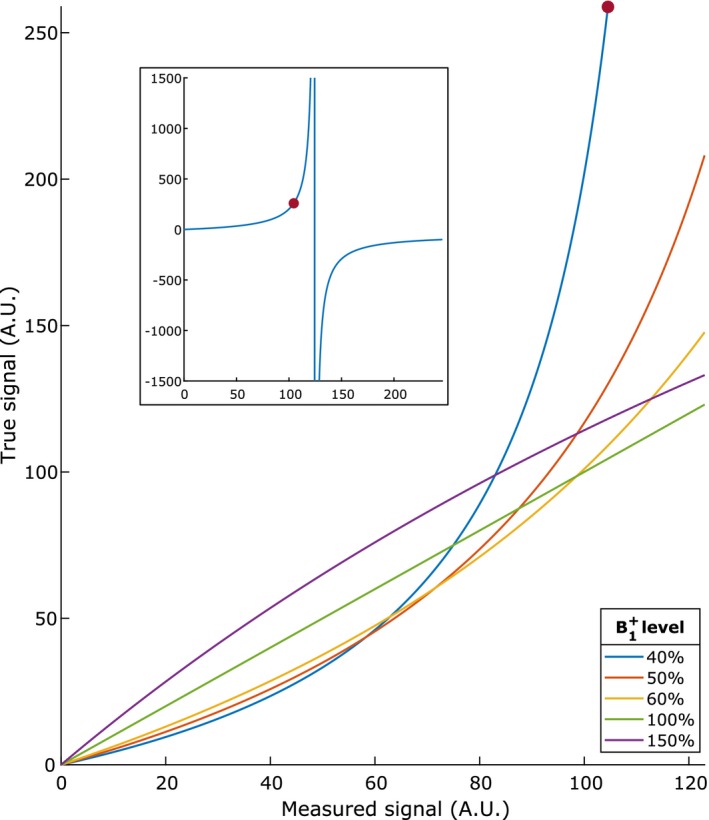
True versus measured image intensity at different $B_{1}^{+}$ levels, assuming a flip angle of 15° and a TR of 5.8 ms as in our patient study (see Section 2.3). This direct mapping is the basis of the proposed correction mechanism. The inset shows the nonlinear behavior of this mechanism for measured image intensity levels higher than the theoretical maximum: the blue curve shows the mapping from measured signals to corrected signals for a $B_{1}^{+}$ level of 40%, and the red dot indicates the theoretical maximum signal

Figure 3 shows a plot of this direct signal intensity mapping for several levels of $B_{1}^{+}$, with a nominal flip angle of 15° and a TR of 5.8 ms as in our patient study (see Section 2.3).

Note that the direct signal intensity mapping of Equation 3 still depends on ρ. In the Haacke‐based correction strategy, a ρ‐map was estimated using the precontrast image, by inverting the signal equation (Equation 1). To avoid introducing a ρ‐map as another source of noise into our correction strategy, we use a single value for the entire scan. Although this is a simplification, the direct mapping of Equation 3 still preserves proton density contrast from the measured image in the corrected image. In most cases, the average estimated ρ in the tumor was used. However, there is a theoretical maximum signal intensity, which is given by $S_{\text{max}}=\rho \;\sin\left(f\theta_{\text{nom}}\right)$. To avoid the highly noncontinuous behavior of Equation 3 that occurs for high values of *M* (see the inset of Figure 3), scans that measured a signal intensity higher than this theoretical maximum were treated differently. For these scans, a heuristic approach was taken: the minimum ρ required to explain the highest measured signal in the tumor was used instead of the average ρ in the tumor. This strategy aims to salvage the data while preserving the measured information on tumor heterogeneity.

### 2.2 Simulations

To investigate the amount of noise amplification of both the Haacke‐based and the proposed direct correction method, a time‐intensity curve was simulated at 100 $B_{1}^{+}$ levels ranging from 1% to 100% of the nominal angle with 1% increments. Gaussian noise was added to the signal such that the SNR was 20, where SNR is defined as average over SD in the precontrast image. Each curve was simulated 3375 times for different noise instances. To investigate at which $B_{1}^{+}$ level the curves no longer benefit from correction because of noise amplification, the SNR and SD of the simulated and corrected curves were calculated per time point, as well as the RMSE between the curves and the ground truth. These values were compared to the RMSE and SNR of the uncorrected simulation, such that the resulting minimum $B_{1}^{+}$ level is independent of the chosen SNR in the simulations. To investigate a T_1_ saturation range wider than is commonly used in clinical DCE protocols, simulations were performed for 6 different flip angles (5°, 10°, 15°, 20°, 25°, and 30°) with a TR of 5.8 ms.^19^, ^20^, ^21^, ^22^, ^23^

### 2.3 Patient data

Data from a previous trial, the PROFILE trial, was used in this work.^24^ This trial included women with histologically proven invasive carcinoma of the breast, eligible for breast conserving surgery based on conventional imaging. This trial ran for 26 months between January 2013 and February 2015. Included patients were scanned using a 7T MRI whole body scanner (Philips Healthcare, Cleveland, OH). Patients were scanned using a bilateral local transmit coil, either in a transceive configuration or with a dedicated 26‐channel receive setup.^14^ A comprehensive multiparametric protocol was performed, including DCE MRI and $B_{1}^{+}$ mapping. DCE MRI with 1 pre‐ and 5 postcontrast (0.1 mmol/kg Gadobutrol, Bayer Schering Pharma AG, Berlin, Germany) images was acquired using a 3D spoiled gradient echo sequence with either TR = 4.3 ms, TE = 2.1 ms, flip angle = 15°, and 1 × 1 × 1 mm^3^ resolution in 108 s per dynamic in case of the transceive setup, or TR = 5.8 ms, TE = 2.5 ms, flip angle = 15°, 0.7 × 0.7 × 0.7 mm^3^ resolution, and SENSE 4 × 2 (left‐right × feet‐head) acceleration in 91 s per dynamic in case of the 26‐channel receive setup.^25^ Binomial pulses in a 1‐3‐3‐1 configuration were used to achieve water‐selective excitation.^26^ $B_{1}^{+}$ maps for 5 coronal slices covering only part of the breast were acquired using the actual flip‐angle imaging (AFI) method with TR1 = 50 ms, TR2 = 250 ms, TE = 1.97 ms, flip angle = 40°, and 5 × 5 × 5 mm^3^ resolution in 44 s.^27^

Out of 55 patients available from the PROFILE trial, 38 were included in the DCE correction. DCE correction on the patient data was only performed using the proposed direct method, because the simulations showed that it outperformed the Haacke‐based method. Reasons for exclusion of patients are outlined in Table 1; the most common reason was insufficient power to generate a $B_{1}^{+}$ map on 1 or both sides. The 38 included patients had a mean age of 61 y (range: 43–74). Based on the surgical specimens, 10 patients had a grade 1 carcinoma, 22 had a grade 2, 3 had a grade 3. The grade could not be assessed post‐surgery in 3 patients, because they had received neo‐adjuvant chemotherapy (NAC).

**Table 1:** Overview of the number of included and excluded patients enrolled in the study

	No. of patients
Patients enrolled in study	55
Patients included in DCE correction	38
Patients not included in DCE correction	17
Patient withdrew from study (imaging not performed)^a^	1
DCE acquisition failure^a^	3
$B_{1}^{+}$ map not performed^a^	1
Insufficient power to generate $B_{1}^{+}$ map on one or both sides^a^	9
DCE imaging artifact (fat excitation)^a^	1
Tumor segmentation failure^a^	2

a Exclusion reason.

Deformable image registration was performed in elastix v4.9 to correct for patient motion between DCE time points.^28^, ^29^ The normalized mutual information similarity metric using 32 histogram bins was optimized with standard gradient descent (1000 iterations, 4096 randomly sampled voxels).^30^ A multi‐scale approach was used with 3 resolution levels. The b‐spline transformation in the final resolution had a grid size of 15 mm.

To eliminate scaling differences between different subjects, histogram normalization was applied to all DCE scans. All intensity values were divided by the estimated noise level of the scan. The noise level was estimated by subtracting a 3 × 3 × 3 box‐filtered precontrast image from the original precontrast image and subsequently determining the SD in a glandular tissue mask. This glandular tissue mask was obtained by taking all voxels above the Otsu threshold of the original (fat‐suppressed) precontrast image.

Even though $B_{1}^{+}$ maps were acquired in this study, they were not always acquired in the same region of the breast as the tumor. Therefore, and because $B_{1}^{+}$ maps are often noisy, we used the template approach developed earlier for a unilateral transmit setup to get the $B_{1}^{+}$ distribution in the tumor.^18^ It has recently been demonstrated that the template approach is also feasible for the bilateral transmit setup used in this work.^31^ $B_{1}^{+}$ template scaling was performed per breast, using the measured $B_{1}^{+}$ maps.

### 2.4 Evaluation of corrected patient data

As was described in Section 2.1, the value for ρ was estimated using either the precontrast image or a heuristic fallback strategy in case the value estimated from the precontrast image was demonstrably too low. We investigated when this fallback strategy was used and whether the values returned by the fallback strategy were in the same range as the values returned by the default strategy. To this end, colored scatter plots were created that indicate which ρ‐estimation strategy was used for different template‐estimated $B_{1}^{+}$ levels in the tumor, as well as the corresponding $B_{1}^{+}$ levels in the measured map and what value of ρ was returned. The $B_{1}^{+}$ level in the measured map was investigated because the AFI $B_{1}^{+}$ mapping strategy used is known to have a reliable linear range limited to 50–150% of the nominal angle.^27^

Curves were quantified by their amount of washin, washout, and peak enhancement. These metrics were calculated per voxel location and defined as follows

(4) $$\begin{array}{c} WO=\frac{S_{1}-S_{5}}{S_{1}}\\ WI=\frac{S_{1}-S_{0}}{S_{0}}\\ PE=\frac{\max_{i=1,\ldots ,5}S_{i}-S_{0}}{S_{0}} \end{array},$$

where *S_i_* is the signal intensity at time point *i*, with time point 0 the precontrast scan, and time points 1–5 the postcontrast scans in chronological order. Note that both washin and washout are calculated using the signal acquired in the first postcontrast scan (*S*_1_).

Tumor masks were obtained semi‐automatically, using the method of Alderliesten et al^32^ with manually determined seed points. The effect of the DCE correction on the curve types was assessed in the most enhancing part of the tumor only.^33^ The most enhancing part of the tumor was selected as those voxels in the tumor mask that showed the highest washin: the top 10 percent of all voxels in the tumor was selected, with no constraints on adjacency. The mean curve in those voxels was calculated and the curve shape was determined: curves that had a washout of more than 10% were designated type III; those with a washout of less than −10% were designated type I; those that fell in‐between were designated type II. The occurrences of each type for both the original and corrected data sets were compared against those found for a set of malignant tumors.^3^

To investigate whether the DCE correction affects tumors of different grades differently, the distribution of washin, washout, and peak enhancement values were calculated inside the tumor mask and plotted for each grade before and after correction. The distributions were calculated using a kernel‐based probability density estimation routine.

## 3 RESULTS

### 3.1 Simulations

**Figure 4:**
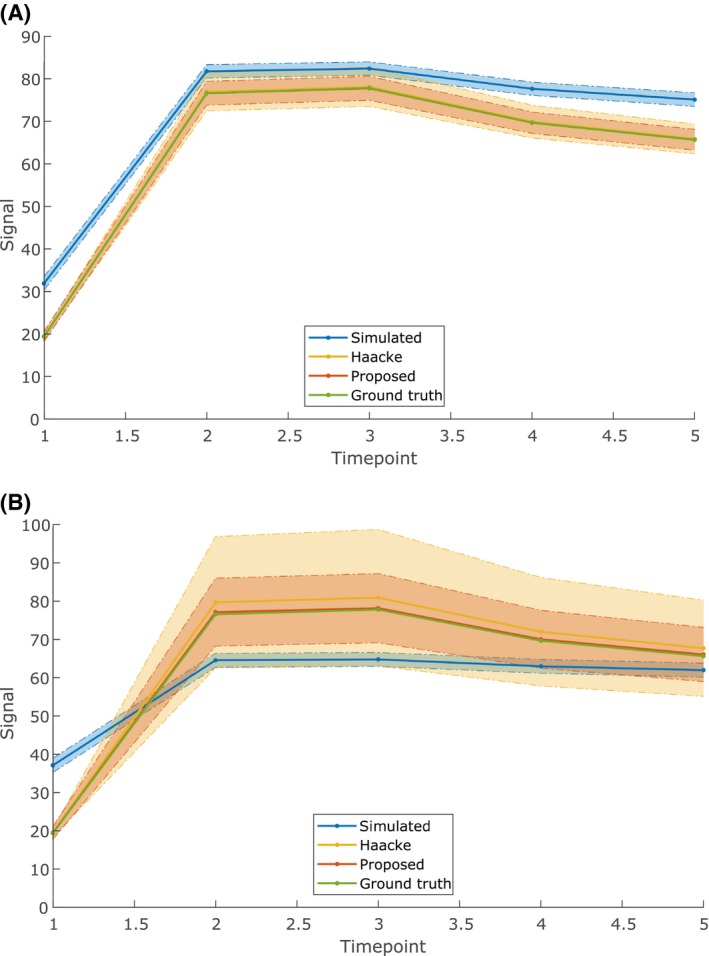
Demonstration of noise amplification in simulations at a $B_{1}^{+}$ level of 50% (A) or 30% (B) of the nominal angle, assuming a flip angle of 15° and a TR of 5.8 ms as in our patient study (see Section 2.3). The time‐intensity curves shown are the mean curve of all noise instances, the shaded area indicates the standard deviation. Noise amplification increases for lower $B_{1}^{+}$ levels for both the Haacke‐based and proposed methods, but the proposed method amplifies the noise less strongly

The noise amplification induced by the Haacke‐based method was consistently larger than that induced by the proposed direct method. Additionally, the mean of the curves corrected by the proposed method was consistently closer to the ground truth. Figure 4 shows this for 2 $B_{1}^{+}$ levels.

Because of the higher noise amplification at relatively low $B_{1}^{+}$ levels, corrected time‐intensity curves at $B_{1}^{+}$ levels below a certain threshold had a higher RMSE with respect to the ground truth than uncorrected ones. For the parameters used in our patient study (flip angle = 15°, TR = 5.8 ms), this threshold was 36% of the nominal angle for the Haacke‐based method and 20% of the nominal angle for the direct method. However, at such low $B_{1}^{+}$ levels, the SNR in the corrected images is very low, below 10% of the original precontrast SNR. The Haacke‐based method maintained the noise level in the corrected images at the level of the original precontrast image for a $B_{1}^{+}$ level of at least 54% of the nominal angle. The direct method maintained this noise level until 43% of the nominal angle. Results for a wider range of flip angles are shown in Table 2. In general, the correctable range becomes wider (extends to lower $B_{1}^{+}$ levels) for increasing T_1_ saturation, and the proposed method consistently returned a wider range than the Haacke‐based method.

**Table 2:** Correctable $B_{1}^{+}$ range for several flip angles assuming a TR of 5.8 ms

Flip angle (°)	Minimum correctable $B_{1}^{+}$ level	
	Haacke‐based method (%)	Proposed method (%)
5	80	77
10	65	57
15	54	43
20	45	35
25	38	29
30	33	25

The correctable range is defined as that range in which correction is still possible without losing SNR with respect to the precontrast image.

### 3.2 Patient data

**Figure 5:**
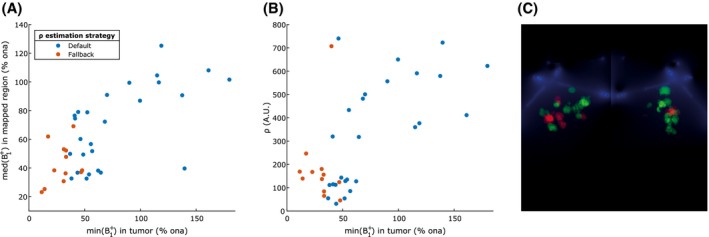
Occurrence and influence of the ρ estimation strategy, indicated by the colored dots. (A) Scatter plot of the minimum $B_{1}^{+}$ level in the tumor (x‐axis) versus the median $B_{1}^{+}$ level in the measured map (y‐axis). The median $B_{1}^{+}$ level on the y‐axis is used as an indicator for the reliability of the measured map, because the linear range of the mapping technique used is limited to 50–150% ona. The fallback strategy is only used for low $B_{1}^{+}$ levels in the tumor (<50% ona). The reliability of the maps did not have an influence. (B) Scatter plot of the minimum $B_{1}^{+}$ level in the tumor (x‐axis) versus the estimated ρ value (y‐axis). The values estimated by the fallback strategy are in the same range as those estimated by the default strategy. (C) Visualization indicating the tumors that were corrected using the default strategy in green and the fallback strategy in red. All tumor positions are shown relative to the position of the coils, indicated in blue. % ona, percentage of the nominal flip angle

As Figure 5A shows, the fallback ρ‐estimation strategy was only used for tumors that have a minimum $B_{1}^{+}$ of 50% or less. The median $B_{1}^{+}$ level of the measured map, and by extent the reliability of those measurements, did not have an influence. Figure 5B shows that the estimated values for ρ by the fallback strategy are in the same range as those estimated by the default strategy. Figure 5C shows the areas where the fallback strategy was applied.

**Figure 6:**
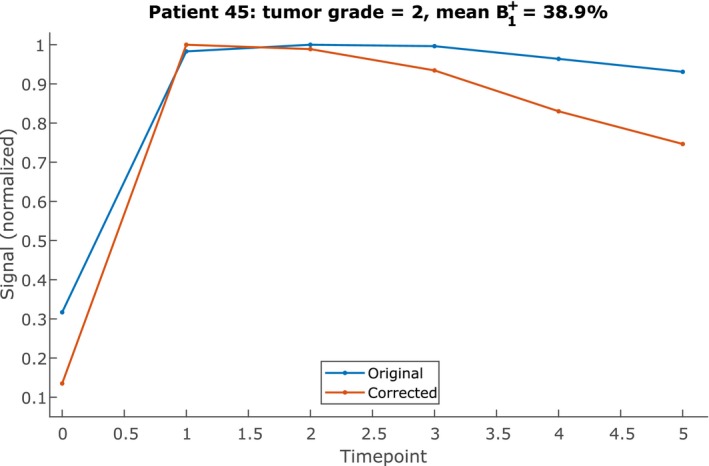
Typical example of an original versus a corrected time‐intensity curve. The curves shown are the mean of the top 10% most‐enhancing tumor voxels. Each curve has been normalized to its own maximum for clarity

Figure 6 shows an example of the effect of correction on the mean curve of the top 10 percent most enhancing voxels of a grade 2 tumor with a low $B_{1}^{+}$ level. This example changed from a type II plateau curve to a type III washout curve. A change to a higher curve type was the most common change across the data set: 5 patients went up from type II to type III, 1 patient went up from type I to type II, and 1 patient went up 2 levels from type I to type III. Only 2 patients changed curve types to a lower type: 1 patient went down from type II to type I, and 1 patient went down from type III to type II. For the majority, 29 of the patients, the curve type did not change. Of all 6 cases that changed into a type III curve, 4 had a grade 2 tumor, 1 had a grade 1 tumor, and in 1 case, the patient received NAC so the tumor grade could not be assessed. Table 3 summarizes the distribution of curve types both of the original and the corrected DCE time series. The distribution of the corrected series corresponds better to the distribution of a malignant group found in literature.^3^

**Figure 7:**
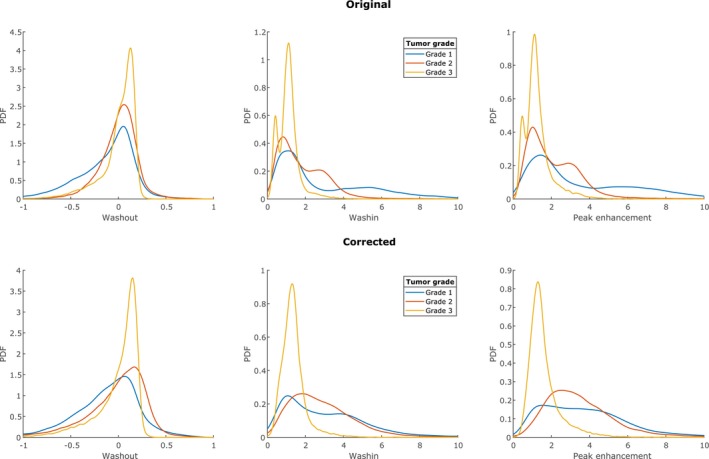
Washin, washout, and peak enhancement distributions per grade, before and after DCE correction. Notice how in all distributions the number of low values are reduced by the correction, as expected

Figure 7 shows the distribution of washout, washin, and peak enhancement values per grade, both for the original and the corrected DCE time series. As expected, especially low values of washout, washin, and peak enhancement are reduced. Additionally, the overlap between the distributions for grade 1 and grade 2 has been slightly reduced.

## 4 DISCUSSION

This paper set out to investigate in which $B_{1}^{+}$ range DCE time series are still correctable for the $B_{1}^{+}$ non‐uniformity effect post acquisition. The answer depends on the amount of SNR one is willing to sacrifice and on the amount of T_1_ saturation that was applied. In DCE‐MRI, the precontrast image usually has the lowest SNR, simply because it has the lowest amount of signal. Therefore, maintaining at least that amount of SNR in the corrected postcontrast images seems like a logical choice. In that case, simulations show the lowest $B_{1}^{+}$ level that can be corrected for with the proposed method is 43% of the nominal angle for the imaging parameters used in our patient study (flip angle = 15°, TR = 5.8 ms). If one is willing to sacrifice more SNR one might go lower, but below 40% of the nominal angle the SNR dropped steeply.

The patient data showed that if one wants to prevent the fallback heuristic ρ‐estimation strategy from kicking in, $B_{1}^{+}$ levels lower than 50% should be avoided. The fallback mechanism is activated when an image intensity higher than the theoretical maximum is measured. This can be caused either by underestimation of the $B_{1}^{+}$ level, underestimation of the value for ρ or a noise‐corrupted measurement. Although it is easy to detect when a signal intensity higher than the theoretical maximum is measured, it is nontrivial to determine which (combination) of the above has caused it. One strategy to deal with this situation would be to throw the measurement away and return either nothing or the theoretical maximum signal in the corrected image. However, this strategy disregards the relative intensity of the affected voxel with respect to its neighbors and consequently all information on tumor heterogeneity is lost. Our strategy tries to heuristically salvage the data by assuming the value for ρ was underestimated. This preserves the measured information on tumor heterogeneity and, as Figure 5B shows, the estimated values for ρ are in the same range as those estimated with the default strategy. Therefore, extending the correctable range down to 43% as suggested in the previous paragraph might be justified. Note, however, that in case the true cause is not the assumed underestimation of ρ, over or under corrections can occur. Both correctable $B_{1}^{+}$ ranges are too narrow to reliably correct all data gathered with the current setup used in this study, but recent results suggest that they may be compatible with the degree of homogeneity that can be achieved with a novel bilateral transmit array.^13^

The effect of DCE correction on the measured time‐intensity curves was as expected. The correction amplified small differences between time points such that most changes in curve type were away from the plateau type II to either type I or type III. The same effect is seen in Figure 7, which shows a clear reduction of low washin, washout, and peak enhancement values. The 1 patient that went down from type III to type II had a $B_{1}^{+}$ level in the tumor of higher than 100%, which explains why the change went the other way.

Although the size of the data set is limited and our findings may be coincidental, they are supported by the fact that after correction the distribution of curve types roughly corresponds to the one found by Kuhl et al^3^ for a set of malignant tumors. Because no ground truth is available for DCE time curves in vivo, this is the only kind of validation that can be performed with the current data set. It is important to consider changes in the inclusion criteria between the patient group used in this study and the one investigated by Kuhl et al.^3^ Both groups only contain malignancies determined on the basis of histology, but an additional inclusion criterion for our group was eligibility for breast conserving surgery.^24^ Most notably, this might cause differences in tumor size, family history and patient age between the compared groups.^34^ A scan rescan protocol where patients are scanned both at 1.5T or 3T and 7T might give a higher evidence level, but even those studies are limited by the fact that DCE‐MRI cannot be performed twice on the same day because of the slow clearance of contrast agents through the kidneys.^35^ Additionally, human reading of curve types might differ from the simple machine classification performed in this work, because the latter only takes the first and last postcontrast into account.

This study has some limitations. First, the correctable $B_{1}^{+}$ range reported in this work, has only been investigated for 6 sets of TR and flip angle. As was reported in Table 2, this range will change depending on the TR and flip angle used. However, most DCE imaging protocols operate with parameters close to those used in this study,^19^, ^20^, ^21^, ^22^, ^23^ probably because these are close to the maximum sensitivity to T_1_ changes (see Figure 2). The correctable $B_{1}^{+}$ range will therefore also be similar. For protocols that have very different parameters, correctable ranges can be found in Table 2 or they can be inferred from simulations like in this work.

A second limitation is that a generic coil‐specific $B_{1}^{+}$ template was the source of $B_{1}^{+}$ information in this work. Although this template performs well, generally as well as conventional mapping approaches, some uncertainty in the level of $B_{1}^{+}$ estimation is to be expected: a previous study reported a mean RMSE of 4% of the nominal angle.^31^ This might be one of the reasons why the proposed method switches to the default strategy only for low $B_{1}^{+}$ levels: an underestimation of the $B_{1}^{+}$ level at low levels could cause the measured signal to exceed the theoretical maximum at the wrongly estimated $B_{1}^{+}$ level. This issue could be solved by using a $B_{1}^{+}$ mapping method with a high dynamic range, but those are time consuming and not likely to be inserted into a clinical protocol. Additionally, our simulations show that the noise enhancement at low $B_{1}^{+}$ levels is considerable, so DCE corrections will probably gain little when a $B_{1}^{+}$ map with a high dynamic range is added.

As proposed by Haacke et al,^17^ a single, fixed value for the precontrast T_1_ was used for all corrections. This value was based on measurements of healthy volunteers. Because most breast tumors are not visible on precontrast DCE MRI, their T_1_ is expected to be in the same range as that of healthy breast tissue. Moreover, the results of Haacke et al^17^ also show that the influence of the fixed precontrast T_1_ is very limited. Still, future studies might investigate if there is a benefit in estimating the precontrast T_1_ on a per‐patient basis.

## 5 CONCLUSIONS

Our results show that for a DCE MRI protocol with a common amount of T_1_ saturation correction for inhomogeneous $B_{1}^{+}$ is feasible at good SNR if the $B_{1}^{+}$ level is at least 50% of the nominal angle. This might be extended down to 43% of the nominal angle, if accurate $B_{1}^{+}$ maps are available at low levels. The effect of correcting a data set with a high variability in $B_{1}^{+}$ was substantial: curve types changed in 25% of the patients, and the distribution of curves across curve types corresponds better with the distribution found in literature after correction.

6

**Table 3:** Distribution of time‐intensity curve types both before and after correction, compared against literature values for a set of malignant tumors^3^

Data set	Type I (%)	Type II (%)	Type III (%)
Kuhl 1999	8.9	33.6	57.4
PROFILE original	13	47	39
PROFILE corrected	11	37	53
